# Heart Failure With Improved Ejection Fraction: Prevalence, Predictors, and Guideline-Directed Medical Therapy

**DOI:** 10.7759/cureus.61790

**Published:** 2024-06-06

**Authors:** Sheethal G Oommen, Ruzhual K Man, Keerthi Talluri, Maryam Nizam, Tejashwini Kohir, Martin A Aviles, Mariana Nino, Lakshmi Gokulnath Jaisankar, Jashan Jaura, Randev A Wannakuwatte, Leo Tom, Jeby Abraham, Humza F Siddiqui

**Affiliations:** 1 Psychiatry, Grigore T. Popa University of Medicine and Pharmacy, Iași, ROU; 2 Research, Lady Hardinge Medical College, Mumbai, IND; 3 Department of Medicine, Ganni Subba Lakshmi Medical College, Rajahmundry, IND; 4 Emergency Department, Valaichennai Base Hospital, Valaichennai, LKA; 5 Cardiology, Amigos del Corazón, Quito, ECU; 6 Medicine, Universidad del Rosario, Bogota, COL; 7 General Medicine, Fathima Institute of Medical Sciences, Kadapa, IND; 8 General Practice, Max Super Speciality Hospital, Bathinda, Bathinda, IND; 9 Surgery, Grodno State Medical University, Grodno, BLR; 10 Internal Medicine, Kowdoor Sadananda Hegde Medical Academy, Mangalore, IND; 11 General Medicine, Yenepoya Medical College, Mangalore, IND; 12 Internal Medicine, Jinnah Sindh Medical University, Karachi, PAK

**Keywords:** guideline-directed medical therapy (gdmt), b-type natriuretic peptide (bnp), remodeling, outcomes, left ventricular ejection fraction (lvef), heart failure with reduced ejection fraction (hfref), heart failure with improved ejection fraction (hfimpef)

## Abstract

Recently, a new category of heart failure with improved ejection fraction (HFimpEF) has emerged in the classification system. This is defined as the subgroup of patients with heart failure with reduced ejection fraction (HFrEF) whose left ventricular ejection fraction has recovered partially or completely, with no specific cut-off values established yet in the guidelines. In our review, we aim to provide an overview of prevalence, predictors, mechanism of remodeling, and management strategies regarding HFimpEF. These patients constitute a sizeable cohort among patients with reduced ejection fraction. Certain patient characteristics including younger age and female gender, absence of comorbid conditions, low levels of biomarkers, and non-ischemic etiology were identified as positive predictors. The heart undergoes significant maladaptive changes post failure leading to adverse remodeling influenced etiology and duration. Goal-directed medical therapy including beta-blockers, angiotensin-converting enzyme inhibitors (ACEIs), and angiotensin II receptor blockers (ARBs) have notably improved cardiac function by inducing reverse remodeling. Despite a more favorable prognosis compared to HFrEF, patients with improved ejection fraction (EF) still face clinical events and reduced quality of life, and remain at risk of adverse outcomes. Although the evidence is scarce, it is advisable to continue treatment modalities despite improvement in EF, including device therapies, to prevent relapse and clinical deterioration. It is imperative to conduct further research to understand the mechanism leading to EF amelioration and establish guidelines to identify and direct management strategies.

## Introduction and background

Heart failure (HF) is a complex clinical condition typically described as a state where the heart's ability to effectively pump or receive blood is compromised. Alternatively, it can be viewed as a dysfunction in the structure or function of the heart resulting in either insufficient output or adequate cardiac output due to compensatory mechanisms like neurohormonal activation and elevated left ventricular filling pressure [1,2]. HF has been recognized as a global health crisis, with an estimated 64.3 million people affected worldwide in 2017 [3]. Cases of HF are expected to continue to rise due to advancing healthcare management resulting in increased survival rates among those diagnosed with HF, owing to new evidence-based treatment modalities and the overall growth in life expectancy. HF also places a significant economic burden on healthcare systems. In the United States alone, the total cost of HF was estimated to be $30.7 billion in 2012 and is projected to increase to $69.8 billion by 2030 [4,5].

HF is diagnosed with a number of laboratory and imaging tests to quantify the degree of dysfunction and to guide treatment. N-terminal prohormone of B-type natriuretic peptide (NT-proBNP) or BNP is a high utility indicator for both the determination of HF severity and the longitudinal monitoring of its trajectory as it shows the degree of cardiac wall stress [6]. Chest radiography is used to assess cardiac dimensions and detect signs of pulmonary congestion along with transthoracic echocardiography (TTE) or cardiovascular magnetic resonance (CMR) to visualize cardiac morphology and estimate the cardiac functional parameters [7,8]. These imaging modalities yield an estimation of the left ventricular ejection fraction (LVEF), which is an integral parameter used for accurate diagnosis and effective management of HF [9].

In 2021, a consensus was reached among the major global scientific organizations to standardize the definition of HF, categorizing heart failure based on LVEF, which is the percentage of blood ejected from the left ventricle (LV) during systole with regard to the end-diastolic volume. The classification system distinguishes between the three main categories: HF with reduced ejection fraction (HFrEF) categorized by an LVEF of ≤ 40%; HF with preserved ejection fraction (HFpEF) characterized by an LVEF of ≥50%; and HF with mildly reduced ejection fraction (HFmrEF), which falls between these two extremities, with an LVEF of 41 to 49%. Additionally, a new category, HF with improved ejection fraction (HFimpEF), characterized by a previously reduced LVEF that has increased to normal or near-normal levels, has been identified [2,10]. This category of HF is associated with a more favorable prognosis compared to HFrEF; however, patients with HFimpEF continue to experience impaired quality of life and pose challenges in clinical management [11].

**Figure 1:**
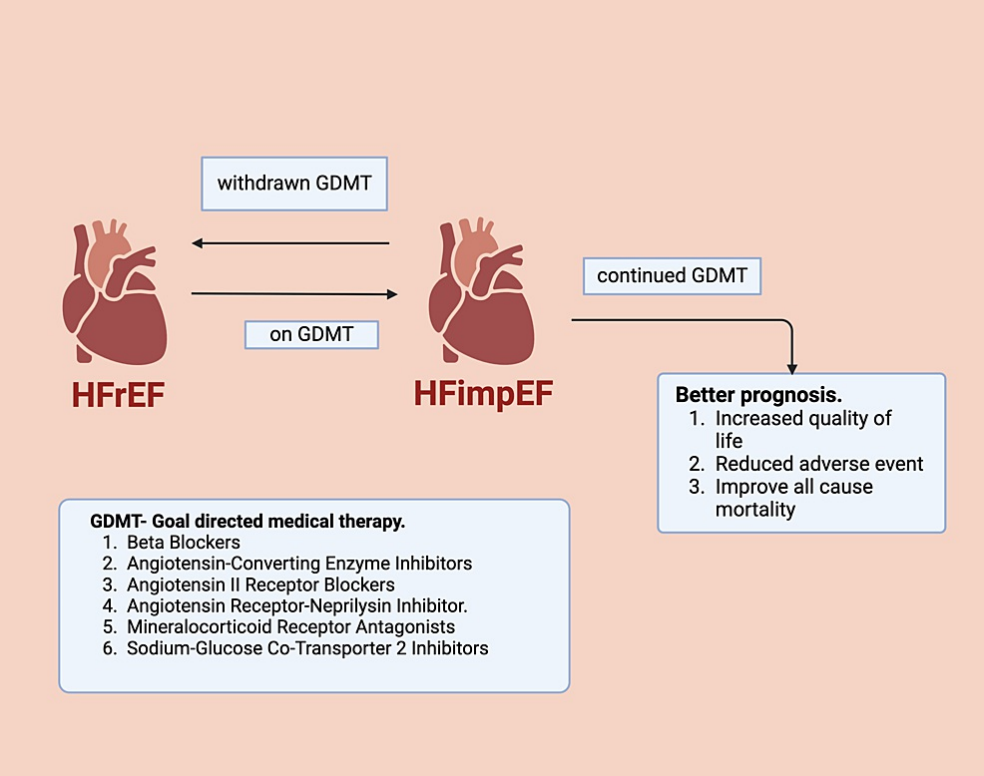
Impact of guideline-directed medical therapy (GDMT) on heart failure with improved ejection fraction (HFimpEF) The figure has been made using biorender.com (Science Suite Inc., Toronto, Canada).

The 2022 American Heart Association, American College of Cardiology, and Heart Failure Society of America (AHA/ACC/HFSA) guidelines recommend continuing guideline-directed medical therapy (GDMT) to avoid relapse and worsening of left ventricular function. However, the clinical management of HFimpEF remains unclear, and further studies are needed to explore how to promote LVEF improvement and improve outcomes [11]. Improvement in the ejection fraction (EF) in patients with HF may be attributed to a process known as reverse remodeling. This is a complex process that takes place due to electrophysiological processes as well as neurohormonal influence [12,13]. HF therapy involving angiotensin II receptor inhibition and beta-blockers is associated with improved LVEF. These neurohormonal therapies act on a cellular level to suppress fibroblast activation as well as normalize G-protein coupled receptor and ryanodine receptor functionality [14-16]. The GDMT for HFimpEF includes optimization of maintenance therapy as well as therapeutic intervention to induce remission of HF. The current management of HFimpEF involves the continuation of the same treatment as prescribed post-stabilization of acute heart failure as there is no clear strategy or guidelines to follow when treating this subgroup of patients with HF. Some of the mainstay treatment options include beta-blockers, angiotensin-converting enzyme inhibitors (ACEIs), angiotensin II receptor blockers (ARBs), neprilysin inhibitors, sodium-glucose cotransporter (SGLT) inhibitors, and diuretics [17-20]. As there is fragmented and minimal evidence to provide definitive guidelines for managing this category of HF, further research encompassing this topic is warranted (Figure 1).

Through this paper, we aim to provide an overview of HFimpEF, including its prevalence, etiology, pathophysiology and predictors, clinical characteristics, and current recommendations for goal-directed medical therapies. By synthesizing existing evidence and highlighting gaps in knowledge, we hope to contribute to a better understanding of this evolving entity and inform clinical decision-making for the management of patients with HFimpEF.

## Review

Definitions and nomenclature

HF is a complex clinical syndrome with signs and symptoms resulting from any structural or functional impairment of ventricular filling or blood ejection. The LVEF is an important factor in the classification of HF as it impacts the prognosis and the therapeutic response. HFimpEF is defined by the American Heart Association as HF with a baseline LVEF ≤ 40%, with a follow-up measurement showing an LVEF >40% [21]. Other authors consider it as a baseline LVEF ≤40%, with a subsequent increase in the LVEF of ≥10% from the baseline and a second measurement of LVEF >40% [22]. Alternative definitions have also been proposed, such as a measurement of LVEF ≥50% preceded by a measurement of LVEF <50% [23]. According to the European Society of Cardiology 2021 guidelines, improved LVEF in a patient is outlined as a history of markedly reduced LVEF (≤40%), with subsequent assessment of LVEF ≥50%. However, this category was not included in the updated 2023 guidelines [24,25].

HFimpEF is associated with better outcomes and prognosis of the disease; however, it does not imply a complete myocardial recovery or normalization of the left ventricular function (LVF) since most of the patient’s cardiac structural abnormalities may persist and LVEF may decrease after treatment withdrawal. This is due to multiple components including the underlying cause, the duration of the disease, treatment compliance, and re-exposure to cardiotoxicity, among others. Therefore, it is not considered a recovered LVEF but rather an improved LVEF [21].

Prevalence of HF

The prevalence of HF is impacted by temporal trends, changes in the identification and burden of risk factors, and the evolution of preventative and management strategies over time. As such, prevalence is usually estimated at the hospital or community level [26,27]. Over 64 million people globally are affected by HF, and current estimates place prevalence at between 1% and 3% of the population. The true prevalence may be higher since these estimates only include patients with recognized or diagnosed HF. In the United States, 5.7 million people are estimated to have HF, and the number is projected to increase to eight million by 2030 [28]. Incidence increases with age, and women make up >50% of patients with HF. The expected increase in prevalence is due to improved treatment options, better survival, and higher overall life expectancy in patients with HF. In high-income countries, where most existing data is from, better management of cardiovascular disease may offset the age-related overall increase in incidence to some extent [24,29]. The incidence of HF approaches 20% in patients over 80 years of age, and the five-year mortality in HF can be as high as 70% [26,27].

Data from several geographic regions, especially South America and Africa, are especially scarce. The available data from various countries also suggests that there may be regional variations in prevalence. In South America, available data estimates prevalence at 1%, while in Australia, it is between 1%-2%, and in South Asia, it may be as high as 6.7% [29, 30, 31]. The estimation of prevalence from the existing literature is also hindered by the non-availability of EF in several data sources, affecting the ability of researchers to categorize HF [29].

Prevalence of HFimpEF

HFimpEF is a relatively newly identified subgroup of patients with HFrEF who have significant improvement in LVEF as a result of management [24,32]. Patients with HFimpEF have been found in several studies to respond better to treatment, and have more favorable outcomes compared to patients with declining or stable EF [22,33,34,35]. Therefore, the estimation of the prevalence of HFimpEF is important to guide management strategies.

The existing literature on HFimpEF places the overall prevalence between 10% and 40% of patients with HFrEF. However, these estimates are rough since definitions of HFimpEF vary in literature and there is significant variability in the etiologies of HF and the management strategies used [33,36-40]. There are significant variations in the recovery of LVEF based on the course and duration of the disease. Patients with stress cardiomyopathy can have high rates of EF recovery, while rates are much lower in patients suffering from chronic HF; rates among patients who have undergone cardiac resynchronization therapy (CRT) are variable [36]. Studies have also found that the incidence of HFimpEF is dependent on the time since the initial diagnosis of HFrEF and declines as time progresses [41-43]. Overall, patients who progress from HFrEF to HFimpEF are more likely to be younger and female and to have HF of nonischemic origin, a shorter disease course, and fewer comorbidities [44-46].

Basuray et al. conducted a prospective cohort study in which HFimpEF was defined as the improvement of EF to ≥50%. A total of 176 (9.67%) of 1821 patients with HF were found to have HFimpEF. They had less severe HF symptoms and less chronic kidney disease than patients with HFrEF and HFpEF, and less hypertension than those with HFpEF. Better event-free survival and biomarker profiles were also associated with HFimpEF than with HFrEF and HFpEF. However, patients with HFimpEF still had significant hospitalizations for HF. Beta-blocker, ACEI, or ARB use was more frequent in patients with HFimpEF and HFrEF than HFpEF [23]. Florea et al. published a study based on data from the Valsartan HF trial. A total of 321 out of 3519 patients (9.1%) with a baseline EF <35% experienced improvement to >40% at 12 months. The improvement in this subgroup was from 28.7% (±5.6) to 46.5% (±5.6). Over 12 months, the change in EF was 17.8±8.0% (ranging from 6%-42%). The difference in EF changes between the subgroups became significant four months after the initial assessment. Subjects with beta-blocker or valsartan use and higher blood pressure (BP) had higher odds of improvement in EF [33].

Agra Bermejo et al. presented that out of 242 patients with LVEF ≤40% (HFrEF), 126 (52%) showed >40 % EF at re-evaluation at an interval of one year. HFimpEF was associated with significantly lower mortality and hospitalization rates, especially in those with EF improvement of >20%. The findings suggest that baseline LVEF and magnitude of recovery may play complementary roles. CRT implantation based on remote LVEF may overestimate the number of people who need it, and the indication for CRT battery replacement in patients with HFimpEF can be questioned. High mortality resulted in a lack of follow-up after one year in a proportion of patients with poor prognosis [47]. Chang et al. performed a study on a community-based sample of middle-aged African-American patients on standard-of-care therapy. HFimpEF was defined as improvement from <35% to >40% in six months. A total of 59 out of 318 patients (18.6%) had HFimpEF, which was associated with lower mortality and fewer hospitalizations. A marked prevalence of LV hypertrophy was noticed. The results may not be generalizable to African-Americans of different age groups from the subjects [48].

Lupon et al. conducted a prospective study in which HFimpEF was defined as an improvement of LVEF from <45% at baseline to ≥45%. A total of 233 out of 940 subjects with baseline EF <45% (24.8%) were found to have HFimpEF at one year. HFimpEF was associated with significantly improved morbidity and mortality relative to HFrEF and HFpEF. The study could not determine if continued evidence-based HFrEF treatment is needed in patients with HFimpEF. A total of 12% of patients with HFimpEF had alcoholic cardiomyopathy. Patients with HFimpEF had significantly lesser time-to-first events and recurrent hospitalizations for HF than those with HFpEF and HFrEF. However, patients were not always seen on their first visit to the HF clinic, and data on revascularizations was not accounted for after the one-year follow-up [45].

Li et al. evaluated an Asian population and looked at LVEF improvement in patients with HFrEF and HFmrEF (mid-range). HFimpEF was defined as ≥10% improvement from a baseline of <50% at six months. A total of 184 out of 447 patients (41.4%) were found to have HFimpEF, and of these, HFmrEF accounted for 71 (38%). The authors noted that if the definition of HFimpEF was changed to absolute values (LVEF improvement from <50% to ≥50%), 231 of the 447 patients (51.6%) would have been classified as HFimpEF [39]. Savarese et al. presented the findings of their study that had no specific EF cutoff values for HFimpEF. Rather, the populations were defined as having HFpEF (LVEF ≥50%), HFmrEF (LVEF 40% to 49%), and HFrEF (LVEF <40%). Transitions from HFrEF to HFmrEF, HFrEF to HFpEF, and HFmrEF to HFpEF were pooled and defined as an increase in EF. Of the total 4942 patients, 18% had HFpEF, 19% had HFmrEF, and 63% had HFrEF at baseline. At follow-up (median time 1.4 years), 25% of HFmrEF patients had transitioned to HFpEF, 16% of HFrEF patients transitioned to HFmrEF, and 10% of HFrEF patients transitioned to HFpEF. Outcomes were more favorable in patients with increased EF (39% lower risk of death or HF hospitalization), and differences in prognosis were most evident in those who transitioned to another category from HFrEF [37].

Ghimire et al. reported a retrospective study, with EF assessed by ≥ two echocardiograms separated by ≥ six months. A total of 3124 patients had EF ≤40% at baseline; of those, 1174 (37.6%) had HFimpEF (defined as absolute improvement ≥10%). These patients were treated with the best medical therapy (ACEIs/ARBs, beta-blockers, mineralocorticoid receptor antagonists (MRAs)). HFimpEF was found to have a substantially better prognosis than HFrEF. Female patients had a lower mortality risk than men after multivariable adjustment, though no differences between the genders were noticed in hospitalization rates [44]. Kalogeropoulos et al. (2016) also performed a retrospective cohort study on outpatients with a three-year follow-up period. HFimpEF was defined as improvement from documented LVEF ≤40% to LVEF >40%. A total of 350 out of 816 (42.9%) patients who were categorized under preserved LVEF at the initiation of the study had improved EF as compared to previous measurements [49].

Nadruz et al. looked specifically at patients with HFmrEF who had improvement in EF. HFmrEF was defined in this study as an improvement from LVEF <40% to between 40% and 55%. A total of 170 out of 944 (18%) patients were found to have HF with improved mid-range EF (HFimpmrEF). Within the midrange LVEF HF population, HFimpmrEF has a more favorable prognosis than HFmrEF. Ventilatory response to exercise was better in patients with HFimpmrEF than HFrEF and similar to HFpEF. The patients also had lower event rates than HFrEF, but they were similar to HFpEF. They also had a lower risk of death, left ventricular assist device (LVAD) implantation, and heart transplantation than HFmrEF and HFrEF. Patients with HFimpmrEF were more likely to have been exposed to chemotherapy in the past. The gain in EF was maintained after a median of 2.8 years of follow-up and recovered LVEF was found to be prevalent in the HFmrEF population. HFimpmrEF patients were more likely to use beta-blockers, ACEIs/ARBs, and CRT or implantable cardioverter defibrillator (ICD) than those with HFmrEF [50]. Trullàs et al. analyzed the patients from the Spanish HF Registry. HFimpEF was described as an LVEF <50% on enrollment but which normalized during follow-up. The study had an older patient population with a mean age of 75. Approximately 25% of patients achieved HFimpEF. However, they were found to still be at risk of death, HF hospitalizations, and to have high levels of natriuretic peptides [51].

DeVore et al. used data from a registry of outpatients with HF (LVEF ≤40%), and changes in health status were assessed as well. A total of 689 of 2092 patients (33%) had ≥10% absolute improvement in LVEF. This was associated with improved health status and reduced risk for future clinical events including all-cause mortality and HF-related hospitalizations. In this study, LVEF assessments were done as part of routine care with a median of 11 months between assessments. Echocardiography was not the modality used in around 20% of patients, but information on parameters other than EF was unavailable [52]. McNamara et al. prospectively evaluated LVEF recovery in post-partum cardiomyopathy through one year postpartum (100 women). By the end of one year, 72% achieved an LVEF ≥50%. A total of 91% with baseline LVEF ≥30% and left ventricular end-diastolic dimension (LVEDD) <6.0 cm recovered. African-American women had more LV dysfunction at presentation and at six months and one year. There was variation in time from postpartum to recruitment in the study. Women of African-American ethnicity usually enrolled later in comparison to other cohorts and had a higher prevalence of hypertension [53].

Kim et al. conducted a retrospective analysis of 90 patients with Takotsubo syndrome. Patients were grouped based on LVEF recovery as either partial (<50%; n=27) or full (≥ 50%; n=63). Partial recovery patients were older and had more comorbid hypothyroidism, levothyroxine use, and longer QT intervals. There were no statistically significant differences in length of stay or adverse events [54]. Singh et al. conducted an uncontrolled prospective cohort study that looked at CRT in 23 patients with chemotherapy-induced cardiomyopathy. The patients were found to have significant improvement in mean LVEF at six months (from 28% to 39%). Chemotherapy may not have been the cause of cardiomyopathy in some patients [55].

These studies faced several methodological challenges and biases that reflected debatable outcomes. A notable limitation of these studies is that EF is a crude marker of cardiac function and remodeling and that no clear consensus exists on the definition of HFimpEF in terms of EF cutoff values. Inferences from these studies are generalizable, but may not be extrapolated to the general population to predict risk. Since there is no consensus on the appropriate treatment regimen for patients with HFimpEF, some of these studies did not analyze the effects of medication dosages and duration and CRT. They also could not identify predictors of CRT response or determine the prognosis of CRT responders since the HFimpEF cohort treated with CRT was insufficient. The true prevalence of HFimpEF may be higher since the patients recruited in most studies were already on optimal therapy. Participants may have been enrolled because of a lack of response to standard therapy and hence may not be representative of clinical practice.

Referral bias was present in some studies, and survival or lead time bias may have had an effect on the outcome. Discrepancies in HF classification and technique variability during echocardiography existed. Only LVEF changes were assessed, and other echocardiographic features were not taken into consideration. LV function was assessed by transthoracic echocardiography instead of three-dimensional (3D) echocardiography or cardiac MRI. Interobserver variability may have led to some misclassification of LVEF based on echocardiography, due to the subjective nature of echocardiography interpretation dependent on the clinician. Some studies were of shorter durations, and the prevalence of improved EF may increase with longer follow-ups or more patients may deteriorate over time. Selection bias may have led to the underrepresentation of patients with severe illness. Some participants in the non-improvement group died or did not present for follow-up echocardiograms, hence the effect of those who did not complete the study cannot be predicted. Potential confounders were not accounted for in certain studies and the authors were not able to rule out the effect of confounding variables despite adjustments. The authors also lacked information on lifestyle factors, which may have influenced recovery. Data was gathered from administrative sources rather than electronic medical records in certain studies. Pre-existing LV problems and biomarkers could not be assessed due to the retrospective design of some studies [37,39,44,45,47-56]. All studies have been summarized in Table 1.

**Table 1:** Prevalence of heart failure with improved ejection fraction NYHA: New York Heart Association; ICD: implantable cardioverter defibrillator; MRA: mineralocorticoid receptor antagonist; ACEIs: angiotensin-converting enzyme inhibitors; ASA: aminosalicylic acid; LVEF: left ventricular ejection fraction; CRT: cardiac resynchronization therapy; CRT-P: cardiac resynchronization therapy pacemaker; CRT-D: cardiac resynchronization therapy defibrillator; RAAS: renin-angiotensin-aldosterone system; HFrEF: heart failure with reduced ejection fraction; HFpEF: heart failure with preserved ejection fraction; CAD: coronary artery disease; PCI: percutaneous coronary intervention; BNP: B-type natriuretic peptide; FDC I/H: fixed-dose combination of isosorbide dinitrate/hydralazine; AF: atrial fibrillation; PVD: peripheral vascular disease; ARNIs: angiotensin receptor-neprilysin inhibitor; IABP: intra-aortic balloon pump; LVEDD: left ventricular diastolic dimension; CKD: chronic kidney disease; GFR: glomerular filtration rate; CCBs: calcium channel blockers; heart failure with midrange and recovered ejection fraction; LV: left ventricle; hs: high-sensitivity; LBBB: left bundle branch block; CABG: coronary artery bypass graft

Author(s) (year)	Article title	No. of patients	Etiology of heart failure	Medical therapy	Definition of LVEF improvement	Percentage of patients with LVEF improvement	Patient characteristics associated with improvement
Berthelot-Richer et al. (2016) [57]	Arrhythmic Risk Following Recovery of Left Ventricular Ejection Fraction in Patients with Primary Prevention ICD	286	Ischemic and nonischemic causes except infiltrative cardiomyopathy, congenital heart disease, long QT syndrome, or inducible ventricular arrhythmias	RAAS inhibitors, beta-blockers, MRAs, diuretics, amiodarone, CRT	Improvement from LVEF ≤35% to >35%	17.1%	Nonischemic etiology; use of CRT devices
Adabag et al. (2017) [58]	Association of Implantable Cardioverter Defibrillators With Survival in Patients With and Without Improved Ejection Fraction: Secondary Analysis of the Sudden Cardiac Death in Heart Failure Trial	2521	Ischemic and nonischemic causes (NYHA II and III)	All subjects had to be on a vasodilator before being randomized to ICD or placebo. Other baseline therapies: MRAs, diuretics, digoxin, and statins	Improvement from LVEF ≤35% to >35%	29.8% in the ICD group; 28.5% in the placebo group	Younger age, less comorbid conditions, and non-ischemic heart disease
Agra Bermejo et al. (2018) [47]	Heart Failure With Recovered Ejection Fraction: Clinical Characteristics, Determinants and Prognosis. CARDIOCHUS-CHOP Registry	449	Ischemic and nonischemic causes except certain infiltrative or restrictive cardiomyopathies, congenital heart disease, and primary right-sided disease	ACEIs, beta-blockers, MRAs, statins, ASA, clopidogrel, oral anticoagulation, ICD	Improvement from LVEF ≤ 40% to >40%	52%	Younger age; better NYHA class; ACEI and beta-blocker use; follow-up for previously implanted ICD; nonischemic etiology
Basuray et al. (2014) [23]	Heart Failure With Recovered Ejection Fraction: Clinical Description, Biomarkers, and Outcomes	1821	Ischemic and nonischemic causes except hypertrophic cardiomyopathy and infiltrative cardiomyopathies	RAAS inhibitors, beta-blockers, MRAs, ASA, digoxin, diuretics, statins, ICD, CRT	Improvement from LVEF <50% to ≥50%	9.67%	Younger age and less hypertension than HFpEF; less ischemic etiology and CAD requiring revascularization than HFrEF; less severe HF symptoms and CKD than HFrEF and HFpEF; more beta-blocker and RAAS inhibitor use than HFpEF
Chang et al. (2018) [48]	Heart Failure With Recovered Ejection Fraction in African Americans: Results From the African-American Heart Failure Trial	318	Ischemic and nonischemic causes	Either FDC I/H or placebo was added to RAAS inhibitors, beta-blockers, MRAs, calcium channel blockers, digoxin, diuretics, or ICD	Improvement from LVEF <35% to >40%	18.6%	Higher baseline LVEF; lower baseline BNP; randomization to FDC I/H treatment; more recent diagnosis of HF; less PVD; less digoxin use
DeVore et al. (2022) [52]	The Association of Improvement in Left Ventricular Ejection Fraction With Outcomes in Patients With Heart Failure With Reduced Ejection Fraction: Data From CHAMP-HF	2092	Ischemic and nonischemic causes	Beta-blockers, RAAS inhibitors, MRAs, ARNIs, CRT, ICD	Absolute improvement of ≥10% in patients with baseline LVEF ≤40%	33%	Female sex; shorter duration of HF; less likelihood of CAD
Florea et al. (2016) [33]	Heart Failure With Improved Ejection Fraction: Clinical Characteristics, Correlates of Recovery, and Survival: Results From the Valsartan Heart Failure Trial	5010	Ischemic and nonischemic causes	Patients were randomized to valsartan. Baseline therapies: ACEIs, beta blockers, MRAs, digoxin, diuretics, ASA, statins, amiodarone	LVEF improvement from <35% to >40%	9.1%	More hypertension; less ischemic heart disease; less severe hemodynamic, biomarker, and neurohormonal profiles; more intense HF medication regimen; higher blood pressure; beta-blocker or valsartan use; less LV dilation; lower hs-troponin T at baseline
Ghimire et al. (2019) [44]	Frequency, Predictors, and Prognosis of Ejection Fraction Improvement in Heart Failure: An Echocardiogram-Based Registry Study	10641	Ischemic and nonischemic causes	RAAS inhibitors, ARNIs, digoxin, beta-blockers, diuretics, MRAs, hydralazine, nitrates, ICD, CRT	Absolute improvement of ≥10% in patients with baseline LVEF ≤ 40%	37.6%	Female sex; younger age; AF; cancer; hypertension; lower baseline LVEF; hydralazine use
Kalogeropoulos et al. (2016) [49]	Characteristics and Outcomes of Adult Outpatients With Heart Failure and Improved or Recovered Ejection Fraction	2166	Ischemic and nonischemic causes	RAAS inhibitors, beta-blockers, diuretics, digoxin, nitrates, hydralazine, warfarin, statins, aspirin, ICD, pacemaker, CRT-D	LVEF improvement from ≤40% to >40%	16.2%	Younger and more likely to be male than patients with HFpEF; less CAD, hypertension, diabetes, chronic lung or kidney disease, and AF than HFpEF; more likely to take a RAAS inhibitor than HFpEF and HFrEF; less likely to have ICD/CRT
Kim et al. (2018) [54]	Patient Characteristics in Variable Left Ventricular Recovery From Takotsubo Syndrome	90	Takotsubo syndrome	Beta-blockers, RAAS inhibitors, CCBs, levothyroxine	LVEF improvement to ≥50%	70%	Younger age, less comorbid hypothyroidism, less levothyroxine use, shorter QT intervals
Li et al. (2021) [39]	Frequency, Predictors, and Prognosis of Heart Failure With Improved Left Ventricular Ejection Fraction: A Single-Centre Retrospective Observational Cohort Study	447	Ischemic and nonischemic causes except corrected congenital or valvular heart disease	RAAS inhibitors, beta-blockers, antiplatelet agents, hypolipidemic agents, ICD, CRT-P, CRT-D, AF ablation, PCI or CABG	Absolute improvement of ≥10% from baseline LVEF <50%	41.4%	Younger age, smaller LVEDD, beta-blocker use, AF ablation, CRT implantation
Lupon et al. (2017) [45]	Recovered Heart Failure With Reduced Ejection Fraction and Outcomes: A Prospective Study	1057	Ischemic and nonischemic causes	RAAS inhibitors, beta-blockers, MRAs, loop diuretics, digoxin, ivabradine, CRT, ICD	LVEF improvement from <45% to ≥45%	24.8%	Nonischemic etiology, absence of LBBB, shorter disease duration, younger age, female sex, lower NYHA class
Madhavan et al. (2016) [56]	Outcomes After Implantable Cardioverter-Defibrillator Generator Replacement for Primary Prevention of Sudden Cardiac Death	253	Ischemic and nonischemic causes except hypertrophic cardiomyopathy, infiltrative cardiomyopathies, primary channelopathies, or congenital heart disease	ICD, RAAS inhibitors, beta-blockers	LVEF improvement from ≤ 35% to >35%	28%	Female sex; lower NYHA class; less beta blocker and RAAS inhibitor use; less PVD; lower serum creatinine levels
McNamara et al. (2015) [53]	Clinical Outcomes for Peripartum Cardiomyopathy in North America: Results of the IPAC Study (Investigations of Pregnancy-Associated Cardiomyopathy)	100	Peripartum cardiomyopathy, excluding patients with significant valvular disease and coronary disease	RAAS inhibitors, beta-blockers, inotropic therapy, IABP	LVEF improvement to ≥50%	72%	Baseline LVEF ≥0.30; baseline LVEDD <6.0 cm; non-African-American
Nadruz et al. (2016) [50]	Heart Failure and Midrange Ejection Fraction: Implications of Recovered Ejection Fraction for Exercise Tolerance and Outcomes	944	Ischemic and nonischemic causes	CRT/ICD, pacemaker, beta-blockers, RAAS inhibitors, MRAs, diuretics, CCBs, anticoagulants, antiplatelet agents, antiarrhythmics, statins	LVEF improvement from <40% to between 40% and 55% (classified as HFm-recEF)	18.9%	Less symptomatic HF; more likely to have exposure to chemotherapy; less diabetes; higher GFR
Ruwald et al. (2014) [59]	Left Ventricular Ejection Fraction Normalization in Cardiac Resynchronization Therapy and Risk of Ventricular Arrhythmias and Clinical Outcomes: Results From the Multicenter Automatic Defibrillator Implantation Trial With Cardiac Resynchronization Therapy (MADIT-CRT) trial	1820	Ischemic and nonischemic causes	CRT-D, antiarrhythmics, RAAS inhibitors, MRAs, beta-blockers, digoxin, diuretics, statins	LVEF improvement from ≤30% to ≥50%	7.3%	Female sex; no prior myocardial infarction; QRS duration ≥150 ms; LBBB; BMI <30 kg/m(2); smaller baseline left atrial volume index
Savarese et al. (2019) [37]	Prevalence and Prognostic Implications of Longitudinal Ejection Fraction Change in Heart Failure	4942	Ischemic and nonischemic causes	RAAS inhibitors, MRAs, digoxin, diuretics, nitrates, platelet inhibitors, oral anticoagulants, statins, beta-blockers, HF devices, CRT/ICD	Transitions from HFrEF (LVEF <40%) to HFmrEF (LVEF 40% to 49%), HFrEF to HFpEF (LVEF ≥50%), and HFmrEF to HFpEF defined as an increase in EF	HFmrEF to HFpEF: 25%; HFrEF to HFmrEF: 16%; HFrEF to HFpEF: 10%	Female sex, less severe heart failure, specialized follow-up for heart failure, nonischemic heart disease but presence of modifiable comorbidities such as anemia and AF, intact renal function
Trullàs et al. (2019) [51]	Heart Failure with Recovered Ejection Fraction in a Cohort of Elderly Patients with Chronic Heart Failure	1202	Ischemic and nonischemic causes	RAAS inhibitors, beta-blockers, MRAs, diuretics, digoxin, aspirin, oral anticoagulants, statins	LVEF improvement from <50% to ≥50%	≈25%	Younger age, less diabetes, better functional status, more ischemic and alcoholic etiology, and more beta-blocker use than HFpEF

Prevalence of HFimpEF in ICD and CRT-Defibrillator (CRT-D) Patients

Madhavan et al. studied ICDs in patients with EF ≤35%. A total of 28% of patients recovered to >35% at generator replacement. However, patients in this group still needed appropriate ICD therapy for ventricular arrhythmias (5% per year). Mortality was similar in patients with EF ≤ 35% and > 35%. Patients who needed ICD replacement for other issues were still included in the study, and this variability may have biased the results [56]. Berthelot-Richer et al. retrospectively analyzed 286 patients who had an ICD placed for primary prevention. Patients were followed for 4.4 years. Recovery was defined as LVEF >35% and was seen in 49 patients (17.1%). Recovery was more likely in patients with nonischemic disease and who had CRT-D. LVEF recovery was associated with a lower arrhythmic risk in the nonischemic cardiomyopathy cohort, but not in the ischemic cohort [57].

Adabag et al. performed a retrospective analysis of patients assigned to placebo vs. ICD. Patients were stratified by EF (≤35% and >35%) at the first repeated measurement after randomization. Of 1273 patients, the repeated EF was >35% in 186 patients (29.8%) randomized to ICD and 185 patients (28.5%) randomized to placebo. The study found that ICD therapy reduced mortality compared to placebo in patients with EF >35%, and they had lower event rates. Patients in this study had to be on an ACEI before randomization but did not have to attain target dosing. Modifications in HF treatment regimen prescriptions had a plausible effect on the results [58]. Ruwald et al. presented patients with CRT-Ds evaluated for LVEF recovery. A total of 7.3% achieved LVEF normalization (>50%). The risk of ventricular tachyarrhythmias was reduced in patients with LVEF >50% and they had a favorable clinical course, with a 7% cumulative incidence of HF or death at three years. However, the risk of inappropriate ICD therapy was still present [59].

Due to the retrospective observational nature of the studies, timeframes could not be ascertained. Other limitations included the possible effects of ICD replacement, the effects of unknown confounders, the absence of a control population, and the lack of information regarding the adequacy of medical treatment. Device programming also varied between patients based on practice with device implantation posing certain challenges [56-59].

Predictors of heart failure with ejection fraction

Clinical Features

We compared several articles in which we found that the most important demographic characteristics in patients with HFrEF that become patients with HFimpEF were age, de novo HF, and gender. Some of these studies also compared race with a p-value in most cases of > 0.05 suggesting it may not have significant relevance [33, 49]. Several studies depicted female gender as a positive predicting factor for improved EF; however, some recent studies have demonstrated that gender may not have significant relevance (p-value: 0.14) [43]. Findings in all studies concurred that patients with HFimpEF were younger than patients with persistent HFrEF or HFpEF (p-value <0.01) [33,43,46,47,49]. Female sex, younger age, atrial fibrillation, cancer, hypertension, lower baseline EF, and using hydralazine are associated with EF improvement of ≥10%. According to various studies, HFimpEF has a higher prevalence among female patients and/or patients without ischemic heart disease [22,33,44].

Regarding physical examination, the characteristics that the articles mentioned were the New York Heart Association (NYHA) functional class, systolic and diastolic blood pressure, basal metabolic rate (BMR), and heart rate. Of these characteristics, patients with HFimpEF had lower heart rate and systolic and diastolic blood pressure than patients with HFrEF or HFpEF. A higher NYHA class, specifically a III or IV, had a worse outcome and a higher all-cause mortality and rehospitalization, but a lower class wasn't a significantly positive predictor for improvement in EF. BMI didn’t have a significant relevance in predicting improvement of EF in patients with HF in recent studies [33,43,46]. Certain findings on ECG including longer QRS complexes, left bundle branch block (LBBB), T peak and T end (TpTe), and QT dispersion (QTD) had a negative predictive factor in correlation to improved EF [60]. ECG is a useful parameter since it is rapid and widely used in clinical situations, but it is not an independent predictive factor. Hence, it should be used in combination with other predictive markers.

Patients with HFimpEF were found to be less likely to have comorbid conditions including hypertension, diabetes, chronic kidney disease, and ischemic heart disease (p-value <0.001) [43, 46]. Cardiac ischemia impairs proper myocardial perfusion, worsening left ventricular function and leading to increased oxidative stress, resulting in the activation of genes such as tumor necrosis factor or interleukin-1β. This, in retrospect, increases left ventricular dilation and worsens the EF, which makes ischemic etiology a negative predictor for HFimpEF [61].

Genetic Markers

The genetic markers that were mostly related to the predicting factors of outcome in HF and improvement in EF were the mutated truncating variants of the titin (TTNtv) gene. A cohort study related to women with dilated cardiomyopathies in peripartum revealed that 83 of the patients who presented with lower EF at the one-year follow-up had a concurrent mutated titin (TTN) gene (p=0.005) [62]. Another study also showed a correlation between TTN mutations and being a male carrier that predicted adverse cardiac outcomes earlier in the disease (9). Both studies concluded that TTNtv mutation can manifest as familial or sporadic dilated cardiomyopathy (DCM), which can result in HFimpEF with the use of guideline-directed medical therapy (GDMT) [22,63]. A mutation in the genes for lamin A/C (LMNA), desmoplakin (DSP) producing gene, sodium voltage-gated channel alpha subunit 5 (SCN5A) gene, and filamin C (FLNC) are genetic markers associated with DCMs that caused a higher risk in sudden cardiac death (SCD) related to malignant arrhythmias [22]. Further investigation is needed to explore a plausible correlation between a mutation in these genes and improvement in EF [64].

Biomarkers

One of the most important biomarkers in heart failure is the natriuretic peptides. The Pro-B Type Natriuretic Peptide Outpatient Tailored Chronic Heart Failure Therapy (PROTECT) study, which analyzed 151 patients with an LVEF <40% with the use of GDMT, revealed that there was an increase in EF of 6.7% and a reduction in systolic and diastolic volumes of 17.3 ml/m2 and 15.7 ml/m2, respectively when N-terminal pro-B-type natriuretic peptide (NT-Pro-BNP) had a value of < 1.000pg/ml [65,66]. Aimo Et al. conducted a meta-analysis using 10 studies with a population of 9289 patients, correlating high-sensitivity Troponin T (hs-TnT) combined with other risk factors to predict all-cause mortality, cardiovascular mortality, and cardiovascular hospitalization. A cutoff value of 18 ng/L for hs-TnT was used, which showed that patients with lower levels had a better outcome and a higher possibility of improving cardiac EF [67]. A study on copeptin revealed that levels higher than 25pg/ml lead to a worse prognosis in children with different cardiomyopathies. On the contrary endothelin-1 (ET-1) at levels of less or equal to 5.9 pg/ml in a study with 115 patients improved prognosis in chronic HF and cardiovascular events. Moving forward these biomarkers will be useful in predicting improvement in EF [68,69].

Some basic laboratory values including renal function tests must be taken into account as biomarkers for EF. Yao et al. conducted a study on 45 patients with chronic heart failure that revealed that elevated levels of uric acid, homocysteine, and cystatin C inversely correlated with LVEF and left ventricular end-diastolic diameter. Higher levels of these renal function tests corresponded with major cardiovascular events and worse outcomes among patients with HF [70]. Similarly, another study highlighted that an increase of > 25% or > 0.3 mg/dl of serum creatinine levels has a deleterious effect on patients with heart failure and can be used as a predictor for cardiac EF. However, cystatin C is a better independent factor for the prediction of outcomes of HF [71]. Glomerular filtration rate (GFR) under 60 ml/min/1.73m2 and or a reduction of >10ml/min/1.73m2 also predicts a worse outcome among patients with HF [72].

Imaging

A two-dimensional (2D) transthoracic echocardiogram was used for the prediction of the likelihood of having a reduced LVEF using global longitudinal strain (GLS). Baseline abnormal GLS of equal or less than 16% had higher odds of predicting a decrease of LVEF > 5% with a sensitivity of 88%, a specificity of 46%, and an accuracy of 0.67 (p<0.001), whereas a > 16% of GLS had higher odds of having a stable LVEF (-5% to 5%) with a sensitivity of 47%, a specificity of 83%, and an accuracy of 0.65 (p=0.002) at follow-up. Hence the study predicted HFimpEF of >50% for each point increase in the absolute GLS [73]. Smaller left ventricular end-systolic dimensions (LVESDs) (3.6 versus 4.8 cm; p<0.01) and higher baseline myocardial systolic performance (9.2% vs. 8.1%; p=0.02) had a correlation as a predictor for HFimpEF in non-ischemic cardiomyopathies. Patients who obtained HFimpEF had 0.8 cm smaller baseline LVEDDs and 1.2 cm smaller LVESDs. Higher radial strain and higher myocardial systolic performance (MSP) were also predictors of HFimpEF [74]. In cardiac magnetic resonance (CMR), late gadolinium enhancement (LGE) is related to myocardial fibrosis. Barison et al. carried out a study for a five-year time period in patients with non-ischemic DCMs (NIDCMs) predicting reversal remodeling and improvement in EF. LGE absence predicted positive outcomes and improvement in LVEF (p=0.043), whereas an LGE equal to or higher than 7% reflected a worse prognosis. A total of 31% of the patients showed reverse remodeling during the median interval of 28 months between the two CMR scans, whereas remodeling was achieved in 51% of the patients at a median follow-up of 42 months [75].

Medications

Ivabradine, bucindolol, and verapamil are a few drugs identified that are used for improving LVEDD, while ivabradine, L-thyroxine, and atorvastatin were noted for enhancing LVESD. Trimetazidine, pentoxifylline, and bucindolol are the drugs that are considered the most effective at improving the New York Heart Association (NYHA) cardiac function score. Lastly, ivabradine, carvedilol, and bucindolol were found to be the top choices for reducing heart rate (HR). These findings highlight varying effects of medicines on heart function [76,77]. SGLT2 inhibitors provide substantial benefits for individuals with heart conditions, irrespective of their cardiac function or treatment environment. They decrease the risk of heart-related deaths, hospitalizations for heart problems, and overall mortality rates. This underscores the importance of considering these medications as primary treatment options for individuals with HF, as they can substantially improve both health outcomes and quality of life. Pharmacological interventions are essential in managing HF, as they impact factors like EF, left ventricular dimensions, and HR. Some medications, like SGLT2 inhibitors, ARBs, and ivabradine, have demonstrated encouraging outcomes in enhancing quality of life, decreasing hospitalizations, and reducing mortality rates among individuals with heart failure [78,79].

Mechanism of remodeling

Post HF, there are two types of remodeling, categorized as adverse remodeling and reverse remodeling. Adverse remodeling leads to deleterious changes in the architecture of the ventricles, worsening the diastolic and systolic function of the heart. Adverse remodeling is seen in males with ischemic etiology and can be predicted with elevated circulatory levels of biomarkers such as BNP and troponin in chronic HFrEF. Reverse remodeling resembles a normal heart that is mostly seen in females with nonischemic etiology with improved EF and lower levels of circulating biomarkers with a lower risk of hospitalization and death [65].

Anatomically, there are two types of remodeling of the heart: eccentric and concentric remodeling. Several cardiac insults can result in eccentric remodeling. For instance, the remodeling in myocardial infarction (MI) can be divided into an early phase and a late phase. Myocyte injury immediately causes an influx of macrophages, monocytes, and neutrophils, which instigates intracellular signaling and neurohormonal activation, initiating a localized inflammatory response. The degradation of interconnected myocytes and collagen causes the infarct to expand, which leads to wall thinning and ventricular dilation and increases the diastolic and systolic ventricular wall stress. Over time the elevated wall stress mediates hypertrophy by triggering mechanoreceptors and renin-angiotensin-aldosterone system (RAAS), which activates the fibroblasts and synthesis of contractile assembly units, laying down new sarcomeres in series, resulting in eccentric hypertrophy. Ultimately, the extracellular matrix forms a collagen scar evenly dispersing the stress on the ventricular wall. However, the weakened wall reduces contraction and the effective systolic function of the left ventricle. Furthermore, aortic and mitral regurgitation also increases the filling pressures of the heart, leading to the activation of similar compensatory mechanisms, resulting in decreased EF. Concentric remodeling is an adaptive response to elevated afterload attributable to conditions including hypertension and aortic stenosis. Increased ventricular wall stress causes arteriolar vasoconstriction, which results in myocyte injury and activation of an intracellular cascade, thus initiating the formation of contractile assembly units. This leads to myocyte hypertrophy and proliferation, and the sarcomeres are added in parallel as a compensatory response, increasing the mass and thickness of the ventricular wall but decreasing the ventricular cavity size. However, the EF is preserved as the left ventricular systolic function is unimpaired. [80].

The heart undergoes two phases of changes after failure. The heart undergoes adaptive changes during the initial response to failure to compensate for the volume and pressure overload, which can be reversed with the removal of hemodynamic stresses. Adaptive changes lead to the thickening of myocytes, an increase in the extracellular matrix and ventricular wall thickness, and a decrease in the size of the chamber. Maladaptive changes occur if there is a persistent fluid overload, which causes irreversible changes that lead to apoptosis and fibrosis of the heart. This pattern of changes causes a decrease in left ventricular wall thickness and an increase in chamber size. These changes cause the function of the heart to decline and cause increased mortality in HF patients [81].

Factors Affecting Remodeling

Diabetes mellitus (DM) is a well-established comorbid condition that can advance atherogenesis and microvascular complications through endothelial dysfunction by downregulating nitric oxide (NO) activity in coronary microvessels. NO plays a crucial role in vasodilation and mediates anti-oxidant, anti-inflammatory, and anti-platelet effects. Diminished activity of NO in coronary vessels leads to deleterious effects on cardiomyocytes, resulting in left ventricular diastolic dysfunction and HFpEF [82]. Even though coronary perfusion therapy decreases the mortality rate of MI patients, new cases of heart failure keep arising. Compensatory mechanisms play a crucial role in maintaining the architectural and functional integrity of the infarcted ventricular wall. However, persistent hemodynamic stress leads to continuous deleterious effects and remodeling. These changes can be noted through a non-invasive technique using cardiac MRI [83,84].

Because of the acute and chronic events of heart failure, there occurs RAAS activation and endothelial dysfunction with ongoing inflammation, leading to oxidative stress and increased collagen deposition with an increase in matrix metalloproteinase (MMP2) and a decrease in tissue inhibitors of matrix metalloproteinase (TIMP), thereby leading to the disordered synthesis of extracellular matrix (ECM) and ECM turnover, which causes tissue remodeling and stiffness of cardiac myocytes fibrosis. These markers such as MMP2 and TIMP serve as a prognostic tool for cardiac function in heart dysfunction. Mineralocorticoid receptors are regulated by microRNAs (miRNAs), which are activated by stress, DM, and hypertension (HTN), which cause cardiac remodeling. Mineralocorticoid receptor antagonists attenuate the remodeling process and improve cardiac function [85]. Studies depicted similar rates of cardiac remodeling between male and female patients reflecting that gender does not have an effect on the remodeling process. Even if the failing heart is treated with appropriate pharmacotherapy, half of the patients undergo a process of remodeling. In the long term, even though there is no difference in outcomes between patients with remodeling and non-remodeling, patients who undergo the remodeling process have a larger rate of hospitalization and increased mortality. Hence, prevention is profoundly advocated in these patient cohorts [86].

Pyroptosis plays a major role in the development of adverse cardiac remodeling. Pyroptosis is an inflammation that acts through caspase 1, caspase 4, and caspase 5 pathways, which act as proinflammatory signals that attract inflammatory cells and cause cardiomyocyte death, fibrosis, and adverse cardiac remodeling. Pharmacotherapy directed against pyroptosis can decrease the adverse effects of cardiac remodeling [87]. In patients with CKD, the accumulation of uremia, decreased vitamin D, increased fibroblast growth factor-23, and elevated phosphorus lead to mitochondrial dysfunction, cardiac maladaptation, myocyte injury, and inflammation, which collectively cause dysfunction and increased remodeling [88]. Remodeling after heart failure has adverse outcomes on patients' lives. Studies have highlighted that induced pluripotent stem cells and embryonic stem cells have the potential ability to be utilized as a regenerative therapy for myocardial muscle damage and can be explored as a promising therapeutic modality in restoring cardiac function among patients of HF. Stem cells induce myocyte differentiation, cardiac muscle maturation, and myocyte regeneration [89]. Innate and adaptive immune systems play a role in ventricular remodeling. Along with the inflammatory process, immunological mechanisms also contribute to the left ventricular remodeling. Pharmacotherapies directed toward the immunological process, such as anti-cytokine, tumor necrosis factor inhibitors, and interleukin inhibitors, help in decreasing the adverse remodeling effect on heart failure patients [90].

Remodeling has adverse outcomes on the heart by decreasing its function and efficacy, but the early initiation of angiotensin receptor-neprilysin inhibitors (ARNIs) has a reverse remodeling effect on heart failure. Beta-blockers and ACEIs reduce the preload and afterload of the heart and improve cardiac function in HF patients; other pharmacotherapies that improve cardiac function are ARBs and mineralocorticoid antagonists. Combination pharmacotherapies, such as ACEIs plus ARBs, ACEIs plus beta-blockers, and ARBs plus beta-blockers, work more effectively than individual therapies [91-93].

There is a difference between cardiac remodeling in patients with HF and without diabetes. In patients with diabetes, there is an ongoing inflammation compared with nondiabetic patients. In diabetic patients, due to insulin resistance and hyperglycemia, there are increased inflammatory cytokines, which lead to fibrosis and remodeling of the heart, thereby decreasing the efficacy and function of the heart compared to nondiabetic patients with HF. Pharmacotherapies such as SGLT2 inhibitors and empagliflozin reduce hyperglycemia and decrease glycolysis, thereby reducing inflammation and fibrosis and decreasing remodeling, which improves the efficacy of the heart. Liraglutide, which is a glucagon-like peptide-1 (GLP-1) antagonist, regulates blood sugar levels, has a cardioprotective function, and causes reverse cardiac remodeling [94,95]. In healthy patients, L-arginine is converted to NO by NO synthase, which leads to the formation of cyclic guanosine monophosphate (cGMP), thus causing vasodilation. NO is impaired in HF dysfunction and causes deleterious effects on heart remodeling. Pharmacotherapies such as riociguat stimulate cGMP and help in heart failure remodeling and dysfunction [96,97].

GDMT for HFimpEF

Rather than traditional treatment methods, targeted therapies specific to individual patient characteristics are more beneficial for improving EF in HF patients, owing to disease heterogeneity [98]. EF is not the best indicator of complete myocardial recovery. Hence, prematurely ceasing patients’ medications is not advised unless complete cardiac recovery is ascertained by further investigation [99].

Clear guidelines for the management of HFimpEF are yet to be published. There is insufficient data on this front. However, drawing out proof from available research, we conclude that the continuation of certain therapies is a necessity for the benefit of the patient [100]. Building on this, it is vital to optimize therapy to achieve required goals and prevent complications by initiating accurate medications, titrating dosages, considering medication switches, or adding new drugs to the regimen if necessary.

Beta-blockers play a key role in improving EF in HF patients regardless of etiology. However, survival rates vary among different beta-blockers, indicating that the mechanism of beta-blockers plays an important role in addition to improving left ventricular function [101]. It has been observed that patients receiving beta-blockers experienced a rise in the average LVEF to 46%. Conversely, when beta-blockers were discontinued in these patients, the LVEF declined to 35%, accompanied by the recurrence of HF symptoms in some cases [36]. Results evaluated from a cohort study showed that amongst patients with HFimpEF who took beta-blockers, regardless of the dose, there was a remarkable reduction in the all-cause mortality risk (hazard ratio: 0.59; 95% confidence interval (CI): 0.40-0.87; p=0.007) [46].

Regarding SGLT2 inhibitors data from the Dapagliflozin Evaluation to Improve the Lives of Patients with Preserved EF HF (DELIVER) trial revealed that patients with HFimpEF had similar rates of complications as did those with an EF that was persistently over 40% during the trial period. It was also found that the patients with HFimpEF stood to benefit from lower rates of complications such as aggravation of HF (HR = 0.84, 95% CI = 0.61-1.14) and death (HR = 0.62, 95% CI = 0.41-0.96) while on dapagliflozin; thus it should be continued even if the ejection fraction has improved [20].

It is no surprise that ACEIs, ARBs, and ARNIs must be continued because of the extensive research done on them. ACEIs significantly reduced deaths due to HF (p<0.0001) and according to a randomized trial of valsartan in chronic HF, it was found to improve EF and clinical condition with p<0.01 [102,103]. The administration of ACEIs contributes to the enhancement of LVEF. However, discontinuing ACEIs in patients with chronic HF results in clinical deterioration and the reversal of beneficial remodeling changes [36].

Sacubitril/valsartan has been observed to enhance LVEF. The primary outcome is defined as the alteration in LVEF from 25.33% at baseline to 30.14% at follow-up after treatment with sacubitril/valsartan (p<0.001). Sacubitril/valsartan has shown remarkable results in improving EF in HF patients, with major improvements observed at higher doses. Additionally, sacubitril/valsartan plays a significant role in reverse remodeling, although it's important to note that patients received optimal medical therapy before initiating sacubitril/valsartan [104].

Patients treated with candesartan plus enalapril plus metoprolol (C+E+M) have shown remarkable improvement in EF and cardiac volumes, as these drugs collectively inhibit various levels in the RAAS pathway and sympathetic nervous system. For C+E+M, the baseline EF was 0.26 ± 0.01. EF was found to increase by 0.05 ± 0.01 with a p-value ≤ 0.001 over 43 weeks, whereas a less significant increase was observed with other combinations of drugs such as C+E, C+M, E+M, or single drugs alone like C or E. Significant improvement was also observed in the end-systolic volume and end-diastolic volume. However, no significant change was observed in reducing BP with C+E+M compared to the other combinations mentioned above. It is important to monitor serum creatinine and potassium levels when administering these combination drugs. This triple therapy is also best tolerated in HF patients, thus showing promising results in patients with HF [105].

Both spironolactone and eplerenone show equal improvement in exercise tolerance, quality of life, and symptoms. However, in patients with HFrEF, the eplerenone treatment group showed an increased LVEF compared to the spironolactone treatment group at rest. In over 12 months of treatment, a remarkable improvement in LVEF was observed in the eplerenone-treated group (40.1 ± 5.7), whereas it was (37.9 ± 3.8± 4.6) in the spironolactone-treated group, with a p-value of less than 0.05. The mechanism of action of eplerenone, effectively blocking the mineralocorticoid receptor and significantly reducing the risk of hospitalization and cardiovascular death, as well as its minimal side effects, contribute to its beneficial outcomes in HF patients [106].

Ongoing research demonstrates potential in novel treatments such as omecamtiv mecarbil and Vericiguat. Omecamtiv mecarbil, a cardiac myosin activator, can increase cardiac contractility, reverse ventricular remodeling, and decrease stress in the ventricular wall, thereby reducing NT-Pro-BNP levels, ventricular volumes, and HR in patients with HFrEF. Based on this, there is potential for a survival benefit with this drug [107]. Vericiguat is another potential drug that is being explored. In HF, the NO-soluble guanylate cyclase (sGC)-cGMP pathway becomes impaired, leading to myocardial injury. Therefore, a treatment such as NO-sGC-cGMP pathway modulation, which facilitates systemic and pulmonary vasodilation, resulting in decreased left ventricular afterload and reversal of left ventricular hypertrophy, may be considered for HFrEF patients, particularly those at increased risk of hospitalization [108].

It is noteworthy that the improvement of EF does not eliminate the risk for ventricular arrhythmias. According to the Sudden Cardiac Death in Heart Failure Trial, the mortality benefit from the use of ICD was the same in patients with a reduced EF and an improved EF [58]. Other research points to the need for consideration of switching from ICD to CRT in those who have an HFimpEF due to reduced occurrences of ventricular tachyarrhythmias and resultant delivery of shock [59]. Perhaps, the need for an ICD must be considered on a case-by-case basis taking into account the underlying etiology such as the presence of genetic condition and residual scar tissue. In any case, further research needs to be done with regard to this matter correlating the etiology of arrhythmia with improved EF [22,109].

Prognosis and outcomes in patients of HFimpEF

Treatment with GDMT has shown improvement in HFrEF and this is known as HFimpEF. Some patients had continued improvement in left ventricular systolic function with the use of GDMT and others with GDMT discontinuation, had a recrudescence of LV systolic dysfunction [110]. HFimpEF patients have a more favorable prognosis compared to patients with HFrEF [36]. Despite an improvement in outcomes, patients with HFimpEF are still at risk for subsequent HF hospitalization and death compared to patients without HF and hence cannot be classified as normal or completely cured from HF [22].

Studies have shown that a shorter duration of HF had a better chance of improving EF compared to those with longstanding HF [52]. HFimpEF has better clinical outcomes and prognosis and lowest mortality when compared with other phenotypes such as persistent HFrEF, HFmrEF, and HFpEF. Patients with HFimpEF needed more catecholamines and mechanical circulatory device support during the initial admission, reflecting more complicated in-hospital circumstances. Ultimately patients with improved EF have a favorable long-term outcome, as compared to grave long-term post-discharge outcomes in HFrEF [46]. The most appreciable improvement in LVEF was seen in the first year after initial diagnosis, with a depreciating trend in LVEF in the 10-15 years after diagnosis [44].

Among 51 patients of the withdrawal of pharmacological treatment for HF in patients with recovered dilated cardiomyopathy (TRED-HF) trial, a substantial decrease in EF within six months of withdrawal of GDMT was noted in nearly half of the patient cohort studied [100]. Hence, the most recent HF guidelines established by the AHA/ACC/HFSA recommend the continuation of GDMT in patients with HFimpEF considering the findings of these studies [20].

Another study reported that 9% of patients recovered their LVEF >50% and normalized their LV end-diastolic dimension on GDMT; 40% of this subgroup experienced a subsequent decline in LVEF, and 5% required heart transplantation or died after 15 ± 4.7 years of follow-up [42]. Studies indicate a positive correlation between younger patients, BMI lower than 22, and lower LVDD to EF improvement up to some extent and better prognosis when given GDMT [49,111]. HFimpEF patients demonstrated lower mortality rates, all-cause hospitalizations, all-cause emergency room visits, and cardiac transplantation or left ventricular assist device implantation compared to patients with persistent HFrEF [22,33,44]. A study reported that nearly 20% of patients with HFimpEF had died or required transplant or ventricular assist device placement by eight years [47]. A long-term follow-up of 174 patients with HFimpEF demonstrated that up to one quarter had subsequent deterioration in their LVEF by eight years and these patients had a five times higher risk of death [49].

Further studies found that HFimpEF is associated with a better biomarker profile, quality of life, and event-free survival compared to HFrEF and HFpEF [46]. However, these patients still experience a significant number of HF hospitalizations, suggesting persistent HF risk [47]. The recovery of systolic function was associated with improvement in HF-related quality-of-life, physical function, satisfaction with social roles, and a reduction in fatigue. A study was conducted in Kansas, United States, to find the correlation between quality of life, physical and social function, and reduction in fatigue with HFimpEF. The study used the Kansas City Cardiomyopathy Questionnaire and other modalities to evaluate patient conditions. Every 10% rise in EF had the Kansas City Cardiomyopathy Questionnaire score improve by a mean (±SD) of 4.8 (±1.6) points (p=.003) [112]. HFimpEF showed a greater reduction in arrhythmic events when EF improved to >50%, whereas EF 30-50% showed a modest reduction. HfimpEF patients on ICD had reduced occurrence, although the risk was not fully eliminated. Therefore, it is advisable to have an ICD if the patient is a suitable candidate [113].

Beta-blockers such as bisoprolol and carvedilol are associated with reduced all-cause mortality risk, with no difference between the two medications [46]. Any substantial improvement in EF is associated with an improvement in survival. Beta-blockers are known to improve EF and long-term survival in HFimpEF [114]. Beta-blockers should be continued even after the restoration of LVEF. Changing patterns of prescription of beta blockers were taken into consideration. A study showed that the patients using beta-blockers at the time of the diagnosis of HFimpEF had a similar prognosis, regardless of beta-blocker prescription at the time of discharge from the index hospitalization [46]. However, other studies have shown that beta-blockers had little long-term benefit in sustaining EF in HFimpEF with EF>50%. Many additional studies have shown that the use of beta-blockers does not have a significant impact on patients with EF >50% and has increased the rate of hospitalization in these patient cohorts [115]. Sacubitril/valsartan should be continued because of the clinical observation that in patients who experience a relapse and recurrent decline in LVEF, there is a higher likelihood of myocyte injury to recur and a decreased ability to recover LVEF the second time around [22]. Patients responded better with RAAS inhibitors, and when the medication was discontinued due to complications like hypotension and acute kidney injury, patients had an increased chance of relapse. Hence, it’s advisable to reintroduce RAAS inhibitors after managing the acute condition to have a better long-term prognosis [115].

## Conclusions

Understanding the complexities surrounding HFimpEF is essential for effective therapeutic management. Biomarkers like natriuretic peptides and troponins serve as crucial predictors, while electrocardiogram parameters and cardiac imaging techniques such as echocardiography and CMR imaging offer valuable diagnostic and prognostic information. 

Management strategies for HFimpEF involve optimizing therapy through GDMTs, including medications like ARNIs, beta-blockers, mineralocorticoid receptor antagonists, and SGLT2 inhibitors. Additionally, novel treatments like omecamtiv mecarbil and vericiguat show promise in improving cardiac function. Continuation of medical therapies involving beta-blockers and ACEIs is crucial for long-term benefits while considering factors such as comorbidities and drug effects on prognosis is essential. ICDs may still be necessary in patients with HFimpEF to manage the risk of arrhythmias, despite improvements in EF.
